# Precision Detection of Salt Stress in Soybean Seedlings Based on Deep Learning and Chlorophyll Fluorescence Imaging

**DOI:** 10.3390/plants13152089

**Published:** 2024-07-27

**Authors:** Yixin Deng, Nan Xin, Longgang Zhao, Hongtao Shi, Limiao Deng, Zhongzhi Han, Guangxia Wu

**Affiliations:** 1 College of Agronomy, Qingdao Agricultural University, Qingdao 266109, China; dyx18863077713@163.com (Y.D.); 18993460419@163.com (N.X.); 2 College of Grassland Science, Qingdao Agricultural University, Qingdao 266109, China; zhaolonggang@qau.edu.cn; 3 High-Efficiency Agricultural Technology Industry Research Institute of Saline and Alkaline Land of Dongying, Qingdao Agricultural University, Dongying 257091, China; 4 College of Science and Information, Qingdao Agricultural University, Qingdao 266109, China; sht@qau.edu.cn (H.S.); denglm68@163.com (L.D.); hanzhongzhi@qau.edu.cn (Z.H.)

**Keywords:** salt stress, soybean seedlings, chlorophyll fluorescence imaging, deep learning, feature fusion

## Abstract

Soil salinization poses a critical challenge to global food security, impacting plant growth, development, and crop yield. This study investigates the efficacy of deep learning techniques alongside chlorophyll fluorescence (ChlF) imaging technology for discerning varying levels of salt stress in soybean seedlings. Traditional methods for stress identification in plants are often laborious and time-intensive, prompting the exploration of more efficient approaches. A total of six classic convolutional neural network (CNN) models—AlexNet, GoogLeNet, ResNet50, ShuffleNet, SqueezeNet, and MobileNetv2—are evaluated for salt stress recognition based on three types of ChlF images. Results indicate that ResNet50 outperforms other models in classifying salt stress levels across three types of ChlF images. Furthermore, feature fusion after extracting three types of ChlF image features in the average pooling layer of ResNet50 significantly enhanced classification accuracy, achieving the highest accuracy of 98.61% in particular when fusing features from three types of ChlF images. UMAP dimensionality reduction analysis confirms the discriminative power of fused features in distinguishing salt stress levels. These findings underscore the efficacy of deep learning and ChlF imaging technologies in elucidating plant responses to salt stress, offering insights for precision agriculture and crop management. Overall, this study demonstrates the potential of integrating deep learning with ChlF imaging for precise and efficient crop stress detection, offering a robust tool for advancing precision agriculture. The findings contribute to enhancing agricultural sustainability and addressing global food security challenges by enabling more effective crop stress management.

## 1. Introduction

Due to the ongoing impact of global environmental changes and agricultural activities, soil salinization has become a major threat to plant growth and crop yield [1,2]. Global statistics reveal that nearly one billion hectares of land are affected by saline–alkali conditions, with almost a quarter of arable land experiencing various degrees of salinization [3]. Furthermore, exacerbated by climate change and unsustainable farming practices, soil salinization in major agricultural regions is escalating, posing a dire threat to food security [4]. Soybean (*Glycine max* L. Merrill) is an important crop rich in oil and plant proteins, with significant implications for human nutrition, animal feed, and oilseed production [5]. Despite soybean being classified as moderately salt-tolerant, soybean seedlings exhibit sensitivity to salt stress, which crucially hinders their normal growth and development, resulting in reduced yield and quality [6]. Therefore, rapid identification research on salt stress during the seedling stage is crucial for ensuring soybean production and agricultural sustainability.

Traditional methods for identifying physiological and biochemical variations in plants typically involve assessing traits such as superoxide dismutase activity, peroxidase activity, malondialdehyde content, and chlorophyll content [7]. Although these methods can accurately indicate the status of salt stress, they have drawbacks such as cumbersome procedures, being labor-intensive and inefficient, and causing significant damage to the plants themselves. In recent years, with the advancement of computer vision and machine learning, there has been a shift towards non-destructive and efficient approaches for studying crop stress. Techniques such as image processing, spectroscopy, and thermal infrared have enabled the extraction of crop stress phenotype features, addressing key issues like stress identification, grading, quantification, and prediction [8]. For instance, Naik et al. [9] compared ten different machine learning classification methods for soybean stress identification, achieving 96% accuracy in soybean identification and proposing a workflow for soybean biotic stress identification. Deep learning is a set of universal machine learning methods, which have been recently successfully and widely applied in many areas to perform all machine learning tasks [10]. The Convolutional Neural Networks (CNNs) represent one of the best deep learning architectures used to recognize, detect, and retrieve content. Deep learning methods, particularly convolutional neural networks (CNNs), have shown significant advantages in complex plant phenotype tasks [11]. In crop stress detection and diagnosis, deep learning methods outperform manual diagnosis. Ghosal et al. [12] focused on soybeans and established an interpretable deep learning model for identifying and grading eight types of biotic stresses in soybeans, achieving a recognition accuracy of 94.13%.

Chlorophyll fluorescence (ChlF) technology, also known as chlorophyll fluorescence induction technology, is a non-destructive method for detecting the photosynthetic status of plants and analyzing their stress resistance. It utilizes excitation light, actinic light, and measuring light to obtain fluorescence intensity values like F_0_, F_0_′, Fm, Fm′, and Fs, which are used to calculate parameters such as photochemical quenching, non-photochemical quenching, and PS II efficiency. Fluorescence imaging systems enable rapid acquisition of fluorescence from cells, leaves, or plants, providing precise image information along with fluorescence parameters or spectra. Chlorophyll fluorescence technology, as a probe for studying photosynthesis, has been widely researched and applied in various fields such as drought [13,14], freezing stress [15], and pathogen and virus stress [12,16]. This technology allows early detection of stress features, aiding in early identification of crop stress. It has been widely used in monitoring and early warning of various crop stress states [17,18]. For example, Liu et al. [19] used dual-band chlorophyll fluorescence imaging technology to screen cucumbers affected by chilling injury, achieving a classification accuracy of 91.4%. Yao et al. [14] integrated dynamic chlorophyll fluorescence with multi-color fluorescence imaging to analyze plant phenotypes. Employing support vector machine, they achieved classification accuracies of 93.3% and 99.1% for distinguishing control plants from those subjected to drought stress for 3 and 7 days, respectively. Similarly, Long et al. [13] utilized chlorophyll fluorescence imaging to assess drought stress in tomato seedlings with high recognition accuracy rates. Osório et al. [20] evaluated chlorophyll fluorescence images of strawberries under iron deficiency, demonstrating its effectiveness for evaluating iron deficiency. Cen et al. [21] used chlorophyll fluorescence imaging to identify HLB disease, achieving the highest classification accuracy of 97% by incorporating average fluorescence parameters and image features.

In this study, chlorophyll fluorescence imaging technology was utilized to capture fluorescence parameters and images of soybean seedling leaves under varying salt concentrations. The study aims to explore the potential application of deep learning and chlorophyll fluorescence imaging technology in plant stress monitoring. By comparing and analyzing the performance of different convolutional neural network models in salt stress recognition, and investigating the impact of chlorophyll fluorescence image feature fusion on classification performance, it provides theoretical and experimental foundations for the development of more effective plant stress monitoring methods in the future. The main contributions of this study are: (1) Realizing rapid detection of salt stress in soybean seedlings using deep learning and chlorophyll fluorescence imaging technology; (2) Comparative analysis of the performance of six classical convolutional neural network models in salt stress recognition; and (3) Exploring the impact of chlorophyll fluorescence image feature fusion on salt stress recognition performance. These findings not only offer valuable insights into salt stress detection in soybean seedlings but also hold significant implications for crop management practices and the development of precision agriculture techniques aimed at optimizing plant health and yield in challenging environmental conditions.

## 2. Materials and Methods

### 2.1. Plant Material and Workflow of Experiment

The genotype of soybean (*Glycine max* L. Merrill) cv. Williams82 was selected for salt stress, and the sterilized seeds were transferred to Petri dishes, germinated in darkness at 25 °C for 4 days within an artificial growth chamber. Subsequently, they were transplanted into plastic pots (15 cm in length, 18 cm in width, and 8 cm in depth, with 14 seedlings per pot) containing Hoagland nutrient solution for hydroponic cultivation and were maintained for 7 days. The hydroponic solution was refreshed every 2 days, and oxygenation was ensured by an air pump. To ensure that the observed plant responses were primarily due to salt stress and not other stressors, the following controls were implemented: (1) Single Variable Control: The hydroponic system was designed so that the salt concentration in the medium was the only variable factor. The plants were cultivated in a climate chamber where other conditions such as light, temperature, and humidity were kept constant. (2) Non-Saline Control Group: A non-saline control group was included as a baseline to compare the physiological changes in plants exposed to different salt concentrations. (3) Repeated Experiments: Experiments were repeated multiple times to ensure the observed effects were consistent and reproducible, minimizing the impact of random or environmental factors.

**Figure 1:**
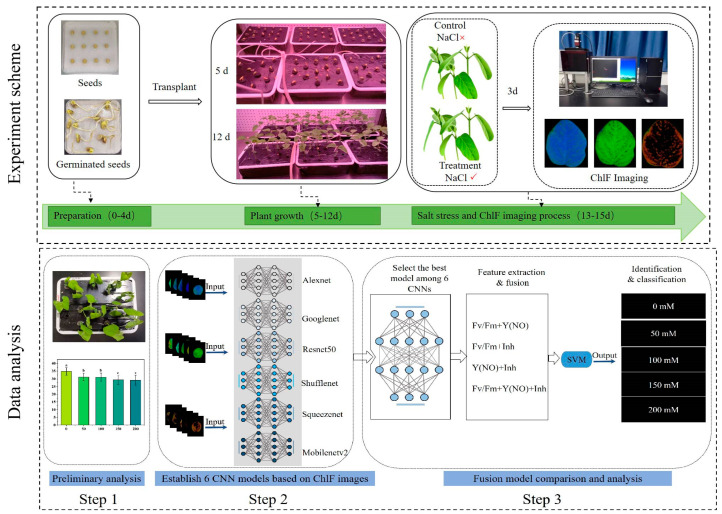
Workflow of using chlorophyll fluorescence imaging to analyze and detect various concentrations of salt stress in soybean seedlings. ChlF: Chlorophyll fluorescence; CNN: convolutional neural network; SVM: support vector machine.

12-day-old seedlings were subjected to salt stress treatment by transferring them into the same hydroponic solutions with varying NaCl concentrations (0 mM, 50 mM, 100 mM, 150 mM, 200 mM) for 3 days [22]. At the end of the treatment, relative chlorophyll contents, expressed as SPAD values, and chlorophyll fluorescence images were acquired from all samples in each group for further analysis. ChlF imaging was used to detect the influence of varying concentrations of salt stress on soybean seedlings by capturing fluorescence parameters indicative of physiological changes under stress conditions. This process included three main steps. Step 1: Preliminary recognition of salt stress utilizing phenotypic characteristics and SPAD values. Step 2: Utilization of six classic CNN networks, namely AlexNet [23], GoogLeNet [24], ResNet50 [25], ShuffleNet [26], SqueezeNet [27], and MobileNetv2 [28], to establish five salt stress recognition models. Model performance was evaluated using a 5-fold cross-validation method. In each fold of cross-validation, 60% of the samples were randomly selected from the dataset to construct the training set, while 20% comprised the validation set, and the remaining 20% constituted the testing set. Step 3: Based on the accuracy results of the previous recognition step, selection of the best CNN model among the six CNNs. Features were then extracted from three types of ChlF images at the same layer, and various feature fusion strategies were employed. Subsequently, fused features were inputted into a support vector machine algorithm (SVM) classifier for further classification and identification of salt concentrations. The parameter values for training the deep learning model are shown in Table 1. The general workflow of the experiment is illustrated in Figure 1.

### 2.2. Chlorophyll Fluorescence Image Acquisition and Augmentation

**Figure 2:**
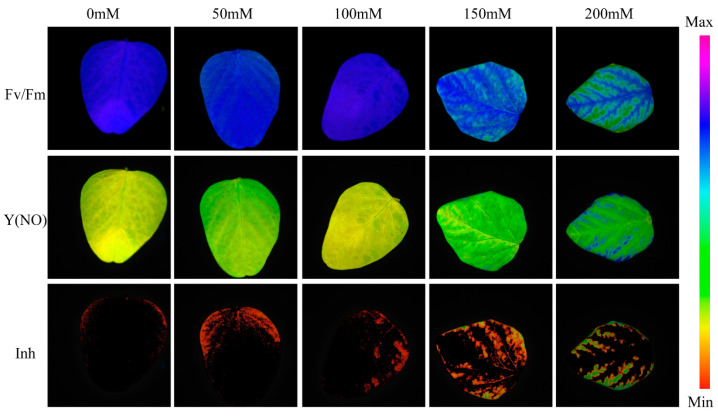
Pseudo-color images of Fv/Fm, Y(NO), and Inh parameters of soybean seedlings under different salt concentration stresses.

In this study, a Chlorophyll fluorescence imaging measurement system (IMAGING-PAM03210218, Zealquest Scientific Technology Co., Ltd., Shanghai, China) was used to collect plants chlorophyll fluorescence images. To evaluate spatial and temporal heterogeneity, four areas of interest (AOI, circle diameter 3 mm) were selected, distributed on both sides of the leaf veins. Before the acquisition of ChlF images, plants were subjected to a dark adaptation treatment for 20 min, which allowed the opening of PSII reaction centers. Subsequently, leaves were placed onto the sample-loading platform, and images of basal fluorescence (F_0_) were captured by applying measuring light pulses (650 nm, red light, 1 μmol·m^−2^·s^−1^), while images of the maximal fluorescence yield (F_m_) were obtained using a saturating red pulse (6000 μmol·m^−2^·s^−1^). Following this, the actinic light (650 nm, 35 μmol·m^−2^·s^−1^) was opened to simulate the ambient light condition, under which the photosynthesis of the plant leaves was activated. The system used ImagingWin2.47 software to collect and analyze data. Figure 2 shows example images of the three fluorescence parameters Fv/Fm, Y(NO), and Inh for each class. These parameters were selected based on their significance in assessing plant responses to salt stress:

Fv/Fm (Maximum quantum efficiency of photosystem II): This parameter indicates the maximum capacity of PSII reaction centers to convert absorbed photons into photochemical energy, reflecting the overall photosynthetic efficiency and health of the plant.

Y(NO) (Quantum yield of non-regulated energy dissipation of PSII): This parameter reflects the efficiency of energy dissipation pathways under stress conditions, providing insights into how plants manage excess energy when exposed to stress.

Inh (Inhibitory effect on PSII quantum yield): This parameter measures the reduction in PSII quantum yield, including Fv/Fm and Y(II), relative to a reference area of interest (AOI), indicating the extent of stress-induced inhibition on photosynthetic performance.

These parameters provide critical insights into the photosynthetic health and stress tolerance of plants under saline conditions, making them essential for understanding the impact of salt stress on soybean seedlings.

We collected a total of 421 ChlF images to create a dataset of leaflets from soybean seedlings subjected to five different salt stress conditions. To expand the dataset, a standard data augmentation scheme was applied, which included transformations such as horizontal and vertical flips, as well as 45°, 90°, 180°, and 270° clockwise rotations. This resulted in an augmented dataset comprising 2947 images. Table 2 presents the distribution of original and augmented ChlF images for each chlorophyll fluorescence parameter across the five salt stress levels.

### 2.3. SPAD Value Determination

At the end of salt treatment, the chlorophyll content of each group was measured using a portable SPAD-502 m (Konica Minolta Investment Co., Ltd., Shanghai, China). The first and second fully expanded leaves of soybean plants were selected for measurement, with SPAD values recorded at three points each on the left, right, and upper sections of the leaves, while avoiding the midribs. The average value of the three SPAD readings for each leaf was calculated to obtain a representative degree of leaf chlorosis.

### 2.4. Software

Image processing and data analysis were conducted using Matlab 2021a (MathWorks, Natick, MA, USA). Matlab was employed for image preprocessing, feature extraction, and integration with the deep learning models used in this study. For graphical displays and result visualization, ORIGIN^®^ (Version 2022, OriginLab Corporation, Northampton, MA, USA) was utilized.

## 3. Results

### 3.1. Changes in Leaf Phenotype and SPAD Values

**Figure 3:**
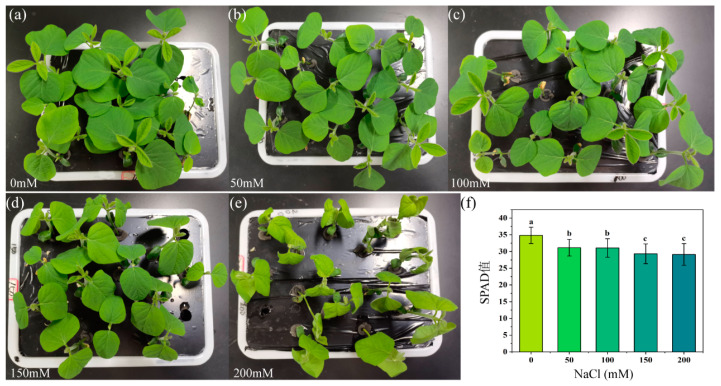
Soybean seedling images and leaf SPAD values under various salt concentration stress. (**a**–**e**): Phenotypic images of soybean seedlings treated with 0, 50, 100, 150, and 200 mM NaCl, respectively. (**f**): SPAD values measured from the leaves of soybean seedlings under different salt concentrations of salt stress treatment. Values shown are means ± SD of three measurements taken in all leaves per treatment. The lowercase letters in the bar charts represent significant differences between the indicated groups as tested with a one-way analysis of variance (ANOVA, *p* < 0.05).

The visible images of soybean seedlings under different salt stress conditions are depicted in Figure 3a–e. No obvious difference can be discerned by the human eye between control and salt-stressed soybean seedlings treated with 50 mM, 100 mM, and 150 mM NaCl solutions. However, under the 200 mM treatment, soybean seedling leaves exhibited slight yellowing and noticeable wilting, accompanied by some curling leaves. Chlorophyll is the most important pigment in crop photosynthesis, and its concentration variation directly affects the health status of crops. The Chlorophyll Meter (Soil and Plant Analysis Development, SPAD) can indirectly estimate the relative content of chlorophyll by measuring the transmittance coefficient of plant leaves. Compared to the untreated control group (0 mM salt concentration), the SPAD values of soybean seedlings decreased significantly with increasing salt concentration. Specifically, at a salt concentration of 50 mM, the SPAD value decreased by 10.51% relative to the control group. When the salt concentration was increased to 100 mM, the SPAD value showed a 10.86% decrease. At a salt concentration of 150 mM, the SPAD value decreased by 15.83%. Finally, at a salt concentration of 200 mM, the SPAD value further decreased by 16.41% (Figure 3f). According to the statistical analysis results of leaf SPAD values, three distinct types can be distinguished: 0 mM and 50 mM form one type, 100 mM and 150 mM constitute another type, while 200 mM represents a separate type.

### 3.2. Salt Stress Recognition Results Using Various CNN Models

**Figure 4:**
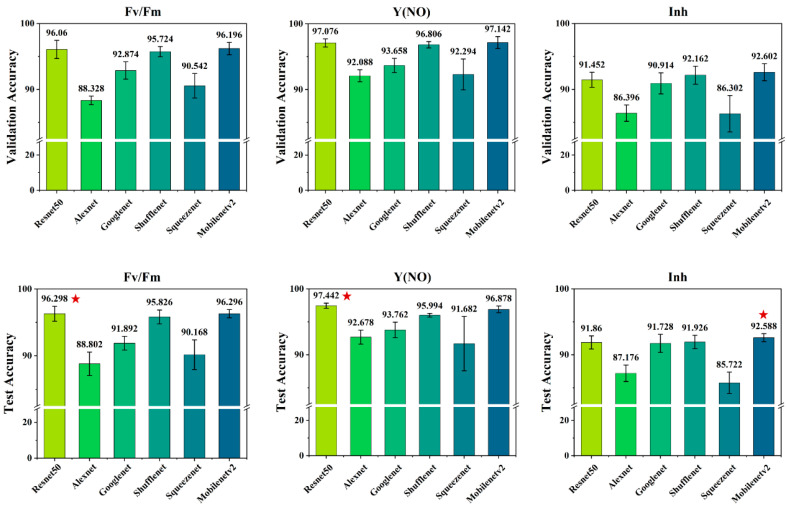
Comparison of validation and testing accuracies among six different CNN models. The red asterisk indicates the highest accuracy.

By separately utilizing three types of ChlF images as model inputs, we employed six convolutional neural networks (CNNs) for salt concentration recognition and established salt stress state discrimination models. The model accuracy of different inputs was analyzed and compared to further select the best CNN model. The salt stress recognition accuracy of each method is shown in Figure 4. The results indicate that among the six CNN models, Resnet50 performed best for the Fv/Fm parameter with test and validation accuracies of 96.298% and 96.06%, respectively, surpassing Squeezenet by 6.13% in test accuracy and 5.92% in validation accuracy. For the Y(NO) parameter, Resnet50 also excelled with test and validation accuracies of 97.442% and 97.076%, respectively, outperforming Alexnet by 4.764% in test accuracy and 4.988% in validation accuracy. Regarding the Inh parameter, Mobilenetv2 achieved the highest test accuracy of 92.588%, exceeding Squeezenet by 6.866% in test accuracy and 6.28% in validation accuracy.

### 3.3. Various Salt Stress Recognition Results Based on Feature Fusion Models

Due to the superior performance of the Resnet50 network in recognizing salt concentration from single-type of ChlF images among six CNNs, we chose it for further feature fusion analysis. Firstly, at the avg_pool layer of the Resnet50 network, features are extracted from the training dataset of the same fold (the 5th fold is chosen for this experiment) of the three types of ChlF datasets. Subsequently, features from two or three types of ChlF are fused. The fused features extracted from the average pooling layer of ResNet50 were fed into a Support Vector Machine (SVM) classifier to distinguish between different salt concentration levels. This approach leverages the combined discriminative features to enhance classification accuracy. The results are presented in Table 3. It is evident that after ChlF feature fusion, the classification results of all four fused models exceeded the recognition accuracy of single type ChlF images (refer to Figure 3). Excitingly, the feature fusion model of three type ChlF images reached validation and test accuracies of 98.37% and 98.61%, respectively. Overall, feature-fused models provided better and more robust results for the accuracy of both the training and validation datasets.

### 3.4. Evaluating Indicator

Although Accuracy is one of the most common evaluation metrics, it can sometimes be deceptive. In the case of imbalanced datasets, the value of Accuracy tends to favor the majority class. Therefore, in addition to evaluating Accuracy, it is necessary to assess more metrics to evaluate model performance effectively. This study introduces metrics such as Precision, Recall, and F1-Score to further comprehensively evaluate the performance of each model. Precision is one of the indicators that can represent the model’s ability to predict correctly. It signifies the proportion of correctly predicted samples out of the total predicted results by the model. Recall, also known as sensitivity, is the proportion of correctly predicted samples out of the total actual samples. F1-Score is a comprehensive evaluation metric that combines Precision and Recall indicators, with values ranging from 0 to 1, where 1 represents the optimal output of the model and 0 represents the worst output. The calculation process of Precision, Recall, and F1-Score is as follows: Firstly, define four basic metrics: samples with actual values as positive and predicted as positive are denoted as True Positives (TP); samples with actual values as positive and predicted as negative are denoted as False Negatives (FN); samples with actual values as negative and predicted as positive are denoted as False Positives (FP); the number of samples with actual values as negative and predicted as negative is denoted as True Negatives (TN). Then, the calculation formulas for Precision (P), Recall (R), and F1-Score are as follows:

(1) $$P=\frac{TP}{TP+FP}$$

(2) $$R=\frac{TP}{TP+FN}$$

(3) $$F1-Score=\frac{2PR}{P+R}$$

These data pertain to the performance of different feature fusion methods in a particular task. As shown in Table 4, four feature combination approaches were considered: pairwise fusion of two features and triple fusion of three features. Each approach was evaluated based on corresponding precision, recall, and F1 score metrics. The results indicate that the triple feature fusion method performs the best across all metrics, exhibiting higher precision, recall, and F1 scores. Specifically, employing the combination of Fv/Fm, Y(NO), and Inh features achieves the highest levels of precision (98.70%), recall (98.57%), and F1 score (98.62%). This suggests that incorporating more features through fusion can enhance the performance of the model in the given task.

### 3.5. Confusion Matrix Analysis

**Figure 5:**
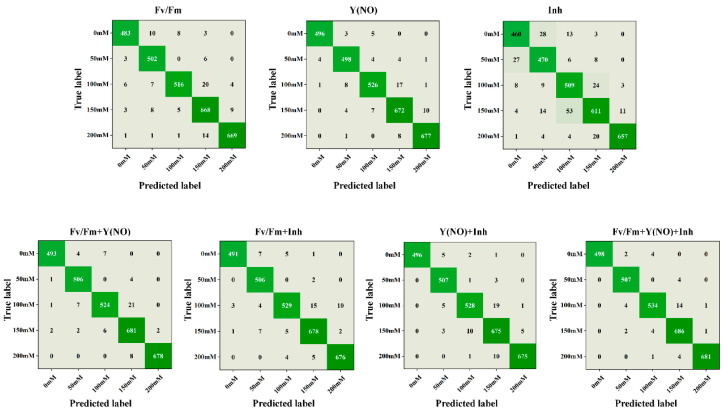
Confusion matrix of different modeling algorithms. The different shades of green represent varying levels of classification accuracy, with darker shades indicating higher accuracy and lighter shades indicating lower accuracy.

Five-fold cross-validation accuracy can reflect the overall modeling performance of different models. However, for classification problems, in addition to the overall model accuracy, the classification performance under different salt concentration stress states can better demonstrate the quality of the model. Therefore, confusion matrix analyses were conducted on the ResNet50 network based on single-type ChlF images and four fusion models based on extracted features of ChlF image, as shown in Figure 5. As shown in Figure 1, each column in the confusion matrix represents the actual attributed class, and the data within each column represent the number of samples in that class. Each row represents the predicted class, and the data within each row show the number of predicted samples in that class. The diagonal values represent the number of correctly classified samples, while off-diagonal values represent the number of incorrectly classified samples as other classes.

When classifying salt concentrations based solely on the Fv/Fm parameter of ChlF images, a high level of concordance between the true and predicted labels is generally observed, indicating favorable performance of the images in salt concentration classification. However, at elevated salt concentrations such as 150 mM and 200 mM, occasional instances of misclassification may occur. Upon incorporating the Fv/Fm and Y(NO) features, a marginal enhancement in classification performance is noted, particularly evident at intermediate concentrations (100 mM and 150 mM), suggesting a discernible role of the Y(NO) feature in distinguishing these concentration levels. Conversely, when employing the Inh feature for classification, a higher incidence of misclassification is observed at high salt concentrations (150 mM and 200 mM), possibly attributed to the suboptimal performance of this feature under such conditions. Finally, the amalgamation of all three features for classification yields an overall improvement in performance, particularly pronounced at high salt concentrations. This underscores the comprehensive information capturing capability of the combined features in ChlF images, consequently enhancing classification accuracy.

### 3.6. UMAP Dimensionality Reduction Analysis

**Figure 6:**
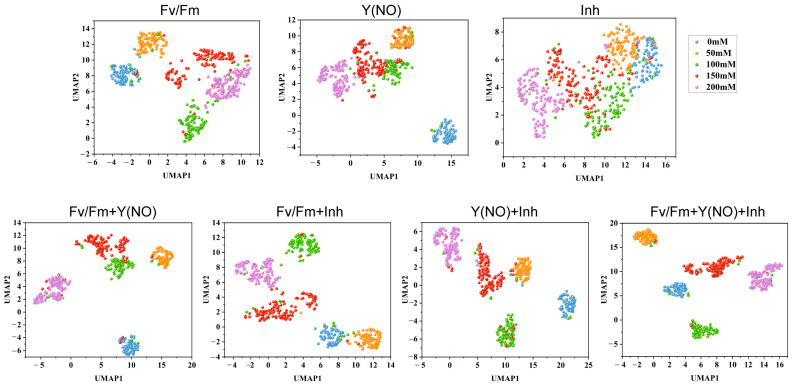
The scatter plot of 2D dimensionality reduction test features using UMAP.

In this study, we performed 2D dimensionality reduction analysis using the UMAP technique on the features extracted from the avg_pool layer of ResNet50 for the same fold of the test set, as well as on the fused features obtained from feature extraction and fusion. The results are visualized in Figure 6, depicting the distribution of test features in a 2D space. Each data point represents a test sample, with its position in the UMAP space reflecting its relative position in the original high-dimensional feature space.

Upon examining the scatter plots of single-type ChlF image features, it was observed that the Fv/Fm features displayed the best distribution, followed by Y(NO), while the distribution of Inh features was the most chaotic. This was evidenced by the intersecting phenomena among different salt concentrations in the scatter plot of Inh features. However, the scatter plot distribution of fused features was notably superior to that of single-type ChlF image feature scatter plots. In the scatter plot of fused features, distinct clustering phenomena were observed, with clear delineation of 5 clusters corresponding to 5 salt concentrations. Moreover, the scatter plot of fused features resulting from the fusion of three types of ChlF image features demonstrated the best performance, with tightly clustered points within each cluster structure and minimal occurrence of crossing or overlapping phenomena between cluster relationships, along with fewer outliers or anomalous points.

## 4. Discussion

In this study, we employed deep learning techniques and chlorophyll fluorescence (ChlF) imaging technology to enhance the detection and understanding of salt stress in soybean seedlings. Our findings reveal significant advancements in both methodology and understanding of plant responses to salinity, which have broad implications for agricultural sustainability and crop management strategies.

### 4.1. Insights into Plant Responses to Salt Stress through ChlF Imaging

The utilization of ChlF imaging in the context of plant salt stress represents a significant advancement in our ability to monitor and understand the physiological responses of plants to saline conditions. Salt stress poses a major threat to global crop production and yield, making it imperative to develop effective monitoring and management strategies. ChlF imaging stands out as a precise and non-invasive tool for assessing plant health under such conditions of stress.

One of the key advantages of ChlF imaging is its ability to provide a wealth of information, including various parameters, quenching curves, and images, enabling researchers to gain insights into the intricate mechanisms underlying plant responses to salt stress [29]. The fluorescence parameters analyzed in this study, particularly Fv/Fm, Y(NO), and Inh, are pivotal for evaluating the impact of salt stress on soybean seedlings. Fv/Fm reflects the maximum potential efficiency of PSII, indicating the overall photosynthetic performance under stress. Y(NO) provides insights into the energy dissipation mechanisms that plants utilize to cope with excess energy. Inh highlights the extent of inhibition on photosynthetic efficiency due to salt stress. Studies conducted by Yuan et al. [30], Awlia M. et al. [31], and Rig et al. [32] have demonstrated the effectiveness of this technique in assessing the impact of salt stress on different plant species, such as cucumber and *Arabidopsis*. These studies have highlighted the significant alterations in photosynthetic efficiency and energy dissipation mechanisms induced by salt stress, as evidenced by changes in parameters like Fv/Fm and ΦPSII. Furthermore, the integration of multicolor fluorescence imaging, as highlighted by Tian et al. [33], expands the analytical capabilities by providing information on primary and secondary metabolic processes affected by salt stress. This integration enhances our ability to detect and monitor salt stress across various plant species, contributing to a more comprehensive understanding of plant responses to salinity.

In addition, our results underscore the critical role of ChlF imaging in early detection and intervention. Early diagnosis of salt stress allows for timely management practices that can mitigate adverse effects on crop yield and quality. This has practical implications for precision agriculture, where real-time monitoring and adaptive management are essential for optimizing resource use and ensuring sustainable crop production. Thus, the application of ChlF imaging represents a powerful approach for studying plant responses to salt stress. By leveraging the advanced imaging technique, researchers can gain valuable insights into the mechanisms underlying salt stress tolerance and develop more effective strategies for enhancing crop productivity and resilience in saline environments. This, in turn, contributes to global efforts aimed at addressing food security challenges in the face of increasing salinity in agricultural lands.

### 4.2. Enhancing Salt Stress Recognition with Integrated ChlF Image Feature Fusion

In this study, we evaluated several classic CNN models for their ability to recognize salt stress in soybean seedlings based on ChlF images. Our analysis revealed that ResNet50 outperformed other models across different types of ChlF images, likely due to its deeper architecture and advanced feature extraction capabilities. This finding aligns with previous research highlighting the robustness of deeper neural networks in complex image classification tasks [25].

One of the significant advancements in our approach was the integration of features from multiple ChlF images, which enhanced classification accuracy compared to using single-type ChlF images alone. This improvement can be attributed to the complementary nature of different ChlF parameters. For instance, Fv/Fm, Y(NO), and Inh parameters collectively provide a more comprehensive representation of the plant’s stress phenotype, enabling better discrimination of salt stress levels. Previous studies have also shown that combining multiple imaging modalities can significantly improve stress detection accuracy in plants [34].

Dimensionality reduction analysis using UMAP visualization further confirmed the effectiveness of feature fusion. The distinct clustering of data points corresponding to different salt concentrations in the UMAP space demonstrated the enhanced discriminative power of fused features, a finding supported by similar applications of UMAP in high-dimensional biological data visualization [35]. Moreover, the use of feature fusion not only improves the accuracy of stress detection but also enhances the robustness of the model in varying environmental conditions. This is particularly important for practical applications in agricultural fields, where environmental factors can vary significantly. The ability to maintain high accuracy under different conditions underscores the potential of our approach for real-world applications.

In contrast, the human eye is only capable of distinguishing two types of soybean seedling images under different salt concentrations, and similarly, SPAD values are limited to distinguishing three types (Figure 3). However, CNNs enabled high-accuracy classification of five salt concentrations based on chlorophyll fluorescence images (Figure 4). This highlights the superior capability of CNNs in discerning subtle variations associated with different salt concentrations compared to human observation and SPAD measurements. Additionally, we used convolutional neural networks (CNNs) to integrate and analyze multiple fluorescence parameter images. This data integration method enhances the model’s ability to distinguish specific responses to salt stress, reducing the likelihood of confusion from other stress effects. It is important to note that SPAD data were not used in the CNN analysis; instead, the purpose of including SPAD data and soybean seedling photographs was to illustrate the comparison between human eye observation, traditional instruments, and CNN classification in recognizing salt concentration levels. This comparison further highlights the advantages of CNNs and the benefits of fusing multiple chlorophyll fluorescence images.

The integration of multiple ChlF parameters not only provides a robust approach to salt stress detection but also underscores the potential of advanced imaging techniques combined with deep learning for precise plant stress monitoring. This methodological advancement can pave the way for developing more effective strategies for crop management and stress mitigation in agricultural practices.

However, it is noteworthy that while Fv/Fm and Y(NO) parameters showed significant discriminatory power in our study, Inh appeared to be less effective in distinguishing between different salt stress levels. From a physiological perspective, Inh reflects the inhibitory effect on PSII quantum yield relative to a reference AOI, but its sensitivity to subtle variations induced by salt stress may be limited compared to parameters directly measuring photosynthetic efficiency and energy dissipation [36,37]. This observation underscores the complexity of physiological responses under stress conditions and warrants further investigation into the specific mechanisms influencing Inh measurements.

### 4.3. Future Directions

In future directions of research, it is observed that most studies are still confined to the analysis of traditional chlorophyll fluorescence parameters [13,38,39,40], with relatively low utilization rates. There is a critical need for further exploration of additional valuable information. Therefore, integrating plant phenotypic trait information and leveraging methods such as mathematical statistics, data mining, and pattern recognition are recommended to fully exploit the advantages of chlorophyll fluorescence technology in characterizing plant photosynthesis. This approach promises a more efficient, accurate, and rapid approach for high-throughput plant phenotypic analysis.

A prevalent issue in current stress studies is the lack of clear stress grading, which hampers subsequent stress management. Future research could address this challenge through two primary avenues: Firstly, most environmental studies currently utilize RGB images combined with chlorophyll fluorescence parameters or plant physiological parameters. It is proposed to establish a classification system for different stress levels based on the characteristics of fluorescence images themselves, such as color, texture, and area of stress regions. Secondly, once the optimal range of environmental factors affecting plant production is determined, automation of identification and environmental control for different stress types could be achieved to meet the practical production requirements for prediction and prevention. Such an approach would facilitate a better understanding of plant responses to environmental stress and improve the efficiency and quality of crop production.

Additionally, in field conditions where multiple stresses can coexist, distinguishing between various stressors remains a challenge. Future research should include specific markers and sensors to detect different stress factors. Integrating real-time sensor data and advanced imaging technologies with our deep learning models could pave the way for developing automated stress detection systems. These systems could be deployed in large-scale agricultural settings, offering continuous monitoring and real-time decision support to farmers.

Another promising direction for future research is the application of transfer learning techniques to improve model performance and generalization across different plant species and environmental conditions. Transfer learning can leverage pre-trained models on large datasets and adapt them to specific tasks, reducing the need for extensive labeled data and computational resources. Furthermore, collaboration with agronomists and crop scientists can facilitate the development of practical applications of these technologies. By integrating domain-specific knowledge with advanced imaging and deep learning techniques, we can create holistic solutions that address the complexities of plant stress and enhance agricultural productivity.

## 5. Conclusions

Our study demonstrates the effectiveness of integrating deep learning with ChlF imaging technologies in advancing crop stress detection and management. Specifically, our developed deep learning framework, utilizing ResNet50 with feature fusion from three types of ChlF images, achieved a high classification accuracy of 98.61% in identifying varying levels of salt stress in soybean seedlings, significantly surpassing traditional methods. This capability enables early identification of salt stress, facilitating timely agricultural interventions such as adjusting irrigation and soil amendments to mitigate potential yield losses. Adopting our methodology could optimize resource use, enhance crop management strategies, and promote productivity and profitability for farmers and agricultural managers. Overall, leveraging these technologies supports more sustainable farming practices aligned with precision agriculture principles, thereby contributing to better management of agricultural resources and sustainable food production.

## Figures and Tables

**Table 1:** Parameter values for training deep learning model.

Model	Layer	Mini Batch Size	Initial Learn Rate	Validation Frequency	Image Input Size
AlexNet	25	32	1 × 10^−4^	64	227 × 227 × 3
GoogLeNet	144	32	1 × 10^−4^	64	224 × 224 × 3
ResNet50	177	32	1 × 10^−4^	64	224 × 224 × 3
ShuffleNet	172	32	1 × 10^−4^	64	224 × 224 × 3
SqueezeNet	68	32	1 × 10^−4^	64	227 × 227 × 3
MobileNetv2	154	32	1 × 10^−4^	64	224 × 224 × 3

**Table 2:** The number of original and augmented images collected in the experiment.

Class	Original Images	Augmented Images	Total
0 mM	72	432	504
50 mM	73	438	511
100 mM	79	474	553
150 mM	99	594	693
200 mM	98	588	686

**Table 3:** Model accuracy under various fusion methods through feature extraction and fusion.

Feature Fused Model	Validation Accuracy (%)	Test Accuracy (%)
Fv/Fm + Y(NO)	97.72 ± 1.01	97.79 ± 0.54
Fv/Fm + Inh	97.52 ± 0.85	97.73 ± 0.51
Y(NO) + Inh	97.76 ± 0.38	97.76 ± 0.35
Fv/Fm + Y(NO) + Inh	98.37 ± 0.63	98.61 ± 0.37

Note: The values presented are the means ± standard deviation (SD).

**Table 4:** Model performance evaluation of the test set.

Measures	Single Type ChIF Images			Fusion of Two Type ChIF Image Features			Fusion of Three ChIF Images Features
	Fv/Fm	Y(NO)	Inh	Fv/Fm + Y(NO)	Fv/Fm + Inh	Y(NO) + Inh	Fv/Fm + Y(NO) + Inh
Precision (%)	96.41 ± 2.57	97.43 ± 1.69	91.76 ± 4.71	97.89 ± 2.06	97.80 ± 2.21	97.90 ± 2.13	98.70 ± 1.67
Recall (%)	96.25 ± 2.63	97.33 ± 1.68	91.86 ± 3.72	97.74 ± 2.24	97.70 ± 2.19	97.78 ± 2.16	98.57 ± 1.85
F1 Score (%)	96.29 ± 1.83	97.37 ± 1.27	91.72 ± 3.17	97.79 ± 1.47	97.72 ± 1.36	97.82 ± 1.49	98.62 ± 1.15

Note: The values presented are the means ± standard deviation (SD).

## Data Availability

Data are available on request.
